# BMI Mediates the Association between Macronutrient Subtypes and Phenotypic Age Acceleration

**DOI:** 10.3390/nu16203436

**Published:** 2024-10-10

**Authors:** Kai He, Tong Xu, Xingxu Song, Jiaxin Fang, Kexin Jiang, Chengxiang Hu, Xue He, Yuchun Tao, Lina Jin

**Affiliations:** Department of Epidemiology and Biostatistics, School of Public Health, Jilin University, Changchun 130021, China; hekai22@mails.jlu.edu.cn (K.H.); tongxu22@mails.jlu.edu.cn (T.X.); songxx22@mails.jlu.edu.cn (X.S.); fangjx21@mails.jlu.edu.cn (J.F.); kxjiang22@mails.jlu.edu.cn (K.J.); hucx22@mails.jlu.edu.cn (C.H.); hexue23@mails.jlu.edu.cn (X.H.)

**Keywords:** macronutrients, BMI mediators, aging, high-quality carbohydrates

## Abstract

**Background:** There is growing evidence that diet and aging are associated; however, few studies have examined the relationship between macronutrient subtypes and phenotypic age acceleration, and the extent to which BMI (body mass index) mediates this association is unclear. **Methods:** This study included 6911 individuals who were 20 years or older and had participated in the National Health and Nutrition Examination Survey. Daily macronutrient intakes were calculated and classified by the quartile of their subtypes. PhenoAgeAccel was calculated as an aging index using nine chemistry biomarkers. Multivariable linear regression and isocaloric substitution effects were used to evaluate the association of macronutrients with PhenoAgeAccel. Mediation analyses were used to examine the mediation role of BMI in the association. **Results:** After adjusting for the potential covariates, the consumption of high-quality carbohydrates (β = −1.01, 95% CI: −1.91, −0.12), total protein (β = −2.00, 95% CI: −3.16, −0.84), and plant protein (β = −1.65, 95% CI: −2.52, −0.78) was negatively correlated with PhenoAgeAccel; the consumption of SFAs (β = 1.77, 95% CI: 0.72, 2.81) was positively correlated with PhenoAgeAccel. For every serving of low-quality carbohydrates/animal protein and other calories replaced by one serving of high-quality carbohydrates/plant protein, PhenoAgeAccel would be reduced by about 25 percent. The ratio between BMI-mediated high-quality carbohydrates and PhenoAgeAccel accounted for 19.76% of the total effect, while the ratio between BMI-mediated total fat and PhenoAgeAccel accounted for 30.78% of the total effect. **Conclusions:** Different macronutrient consumption subtypes are related to PhenoAgeAccel, which is partially mediated by BMI, depending on the quality of macronutrients. Replacing low-quality macronutrients with high-quality macronutrients might slow aging.

## 1. Introduction

An increase in life expectancy and a decline in fertility contribute to population aging around the world. By 2050, the elderly population around the world will account for one in six [1]. There is an increased risk of many chronic diseases associated with aging [2,3]. From an economic standpoint, in the next 50 years, aging delayed by 2.2 years will save USD 7 trillion [4]. Previous research has shown that aging may cause a loss of muscle quality [5], neurodegeneration [6], cardiovascular homeostasis, and metabolic disorders [7], and it may affect every organ in the human body. It is important to note, however, that biological aging states are not accurately reflected by chronological age (CA) [8]. It has been proven that PhenoAgeAccel, a new measure of biological aging, permits the identification of morbidity and mortality risks among different subpopulations in the U.S., particularly those who are healthy and disease-free [9].

Aging, as a complex process, is related to physiologic and lifestyle factors, including genetic factors [10], tobacco smoking [11], heavy alcohol drinking [12], weight across adulthood change [13], and diet [14,15,16]. Throughout the course of life, it is believed that dietary patterns play a key role in human health [17] and might lead to groups of individuals with diverse cultural backgrounds having various health outcomes, potentially affecting their dietary patterns [18]. Obesity, for example, is associated with a shorter lifespan. Meanwhile, healthy dietary patterns, like the Mediterranean diet, could promote longevity and reduce the risk of diseases associated with aging [19]. The quality and subtypes of foods and macronutrient intake, which contains distinct food sources of protein [20], carbohydrates, and fat [21], may affect aging. An adequate supply of protein may slow down age-related muscle loss, which may cause a decline in physical function [22,23].

Despite previous studies having reported that macronutrient intake was associated with aging, the result was not entirely consistent. It was found that a low intake of protein and fat was correlated with the prevalence of frailty and sarcopenia [24,25]. Some prospective observational studies have proven that the impact of total fat and carbohydrate intake was not significant [26,27]. Meanwhile, some studies found that a significant relationship only exists in some special subgroups [28]. One of the reasons for the ambiguous results obtained may arise from the failure to take the competitive relationship between the different subtypes of macronutrients into account.

Therefore, in our study, we explore the relationship between the quality and quantity of macronutrients and PhenoAgeAccel using data from the National Health and Nutrition Examination Survey (NHANES). Given that dietary factors are essential for the prevention and management of obesity, we further analyzed the mediating effect of BMI.

## 2. Methods

### 2.1. Study Population

The research draws on data collected from the NHANES, which employs a stratified, multi-stage approach to ensure accurate representation. The details were described earlier [29]. The study included 17,132 non-pregnant NHANES 2005–2010 respondents over 20 years of age who were excluded from phenotype age data loss (n = 9698), diet data loss (n = 281), and extreme energy intake (<800 kcal per day or >4200 kcal per day for males and <500 kcal per day or >3500 kcal per day for females, n = 242). A total of 6911 participants were finally included (Figure S1). Before collecting data, it was authorized by the National Health Statistics Center Institutions Censors and the written knowledge and agreement of each participant [30].

### 2.2. Phenotypic Age Acceleration

PhenoAgeAccel was computed in accordance with the formula of Morgan E. Levine et al. [31]. It was determined using chronological age (CA) and nine biomarkers [32] (i.e., creatinine, albumin, [log] C-reactive protein, glucose, alkaline phosphatase, lymphocyte percent, red cell distribution width, mean cell volume, and white blood cell count). When measured after taking into account chronological age, PhenoAgeAccel is the representation of phenotypic ageing. If a person is PhenoAgeAccel ≤ 0 (>0), they are phenotypically younger (older) than would be expected on the basis of their CA [31].

PhenoAgeAccel was calculated according to age and nine biomarkers, as shown below:

$$\mathrm{Phenotypic}\;\mathrm{Age}=141.50+\frac{ln\{-0.00553\times ln(1-mortalityrisk)\}}{0.090165}$$

where mortality risk = 1 − exp ($\frac{-1.51714\times \exp(\mathrm{xb})}{0.0076927}$) 

and

$$\begin{array}{l} \mathrm{xb}=-19.907-0.0336\times \mathrm{albumin}+0.0095\times \mathrm{creatinine}+0.1953\times \mathrm{glucose}+\\ 0.0954\times \ln(C—\mathrm{reactive}\;\mathrm{protein})-0.0120\times \mathrm{lymphocyte}\;\mathrm{percentage}+0.0268\;\times \\ \mathrm{mean}\;\mathrm{cell}\;\mathrm{volume}+0.3306\times \mathrm{red}\;\mathrm{blood}\;\mathrm{cell}\;\mathrm{distribution}\;\mathrm{width}+0.00188\;\times \\ \mathrm{alkaline}\;\mathrm{phosphatase}+0.0554\times \mathrm{white}\;\mathrm{blood}\;\mathrm{cell}\;\mathrm{count}+0.0804\times \;\\ \mathrm{chronological}\;\mathrm{age} \end{array}$$

PhenoAgeAccel = Phenotypic Age − Age

### 2.3. Dietary Assessment

Diet data were obtained through two non-continuous interviews regarding 24 h dietary recall. The initial dietary recall interview took place at the Mobile Examination Centre (MEC), followed by a second interview by telephone within 3 to 10 days. We averaged two 24 h dietary recalls. Energy intakes and dietary nutrients were assessed from the United States Department of Agriculture’s (USDA) Dietary Research Food and Nutrient Database [33]. The Food and Nutrient Database for Dietary Studies contains information on foods and beverages listed according to the user guidelines based on the Food Patterns Equivalents Database (FPED) and is divided into 37 food pattern components [34]. The study derived a total of 6 macronutrients by grouping similar food types into subtypes, including carbohydrates (high-quality carbohydrates and low-quality carbohydrates), protein (animal protein and plant protein), and fat (unsaturated fatty acids (USFAs) and saturated fatty acids (SFAs)). The detailed grouping is provided in Table S1. Food sources of fats are not examined due to their similarity with protein food sources, and the majority of available evidence on fats focuses on fatty acid types rather than food origins [35]. In our study, participants were classified according to the quartile of their daily macronutrient subtypes (Table S5).

### 2.4. Covariates

We considered gender (male or female), race/ethnicity (non-Hispanic white or others), PIR (≤1.0, >1.0), drinking (yes or no), smoking status (yes or no), energy intake (kcal/day), moderate or vigorous exercise (yes or no), and whether overweight or not covariates. In the NHANES, sociodemographics (age, gender, race/ethnicity, and PIR) and lifestyle behaviors (smoking, drinking, and physical activity) were collected using a questionnaire.

### 2.5. Statistical Analysis

According to the NHANES analysis guidelines, weights of dietary samples, stratification, and clustering were incorporated into the analysis to account for complex survey designs. Macronutrients are divided into quartiles. Demographic characteristics, anthropometric values, and dietary intake are incorporated as the weighted percentage of categorical variables (95% CI) and the mean of continuous variables (95% CI). The relationship between macronutrients and PhenoAgeAccel was examined using multiple linear regression models. The nonlinear relationship between macronutrients and PhenoAgeAccel was examined using restricted cubic splines, providing β and 95% CI. The model was adjusted for sex, race/ethnicity, PIR, whether overweight or not, physical activity, drinking and smoking status, and energy intake. When discussing macronutrients, the isocaloric substitution effect of reducing 1 serving of low-quality carbohydrates, animal protein foods, or 1 g SFAs while increasing 1 serving of high-quality carbohydrates, plant protein foods, or 1 g USFAs was assessed. The detailed method has been described previously [5]. We also used the “BruceR” package in R statistical software to further examine the mediating role of BMI in the relationship between macronutrient intake and PhenoAgeAccel.

Our study uses a stratified analysis as our sensitivity analysis to verify the robustness of the results. The stratified analysis was further based on potential confounding factors, including gender (male or female), race/ethnicity (non-Hispanic white or others), PIR (≤1.0, >1.0), drinking (yes or no), smoking status (yes or no), moderate or vigorous exercise (yes or no), and whether overweight or not. At the same time, we examined the interaction between dietary scores and subgroup variables using the survey’s weighted Wald F statistic.

The statistical analyses were conducted using IBM SPSS Statistics 25.0 (IBM, Asian Analysis Shanghai) and R (version 4.2.3; R Core Team), with all tests being two-tailed. A significance level of a *p*-value < 0.05 was used to determine statistical significance.

## 3. Result

### 3.1. Participant Characteristics

Table 1 presents the characteristics of the study participants stratified by PhenoAgeAccel. In addition to sex and low-quality carbohydrate consumption, the sociodemographic characteristics of the participants differed significantly due to PhenoAgeAccel status. Participants who were non-Hispanic whites had a higher household income, were smokers, were overweight, did not drink alcohol, had lower physical activity, had a lower intake of high-quality carbohydrates, had a lower intake of plant and animal protein, and had lower intake levels of SFAs and USFAs were more likely to experience aging. The total energy intake of PhenoAgeAccel > 0 was significantly higher than that of PhenoAgeAccel ≤ 0.

### 3.2. Association of Macronutrients with PhenoAgeAccel

Table 2 displays the results of the linear regression analysis. In the model with adjustments for age and race, the consumption of high-quality carbohydrates (β = −1.87, 95% CI: −2.74, −0.99), total protein (β = −2.74, 95% CI: −3.87, −1.61), and plant protein (β = −1.96, 95% CI: −2.87, −1.05) was negatively correlated with PhenoAgeAccel. The consumption of SFAs (β = 1.54, 95% CI: 0.48, 2.60) was positively correlated with PhenoAgeAccel. Multivariate linear regression models were used to further adjust for other possible confounding factors. The correlation remained significant, and the highest quartile of the multivariate-adjusted model was (β = −1.01, 95% CI: −1.91, −0.12); the total protein was (β= −2.00, 95% CI: −3.16,−0.84), plant protein was (β = −1.65, 95% CI: −2.52, −0.78), and SFAs were (β = 1.77, 95% CI: 0.72, 2.81). Through further application of the restricted cubic spline, we found that high-quality carbohydrates and PhenoAgeAccel had a nonlinear relationship (P for nonlinearity = 0.001) (Figure S2).

Food sources of high-quality carbohydrates and plant protein are analyzed in Table S2. Tomatoes (β = −0.93, 95% CI: −1.56, −0.30) and other red/orange vegetables (β = −2.36, 95% CI: −3.70, −1.02), refined grains (β = −0.19, 95% CI: −0.70, −0.30), nuts (β = −0.20, 95% CI: −0.36, −0.04), and soybeans (β = −1.03, 95% CI: −1.65, −0.41) were negatively correlated with PhenoAgeAccel. There was no significant difference between PhenoAgeAccel and other types of food.

### 3.3. Isocaloric Substitution Effects

Table 3 shows the isocaloric substitution effect of macronutrient subtypes on PhenoAgeAccel. In totality, we found that reducing low-quality carbohydrates by one part per day, increasing high-quality carbohydrates by one part per day (β = −0.25, 95% CI: −0.45, −0.05), reducing animal protein by one part per day, increasing plant protein by one part per day (β = −0.17, 95% CI: −0.34, −0.002), and reducing 1 g of SFAs and replacing it with 1 g of USFAs (β = 0.06, 95% CI: −0.10, −0.02) were negatively correlated with PhenoAgeAccel.

### 3.4. Macronutrients and PhenoAgeAccel: The Mediating Role of BMI

**Figure 1:**
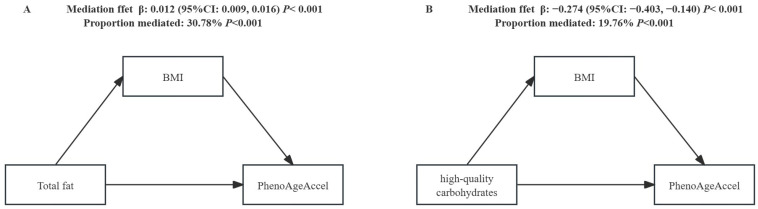
The mediation effect of BMI in the relationship between macronutrient subtypes and PhenoAgeAccel. Adjusted for sex, race/ethnicity, PIR, smoking status, drinking alcohol, energy intake, and physical activity. The indirect effect represents the effect passing through the mediator (BMI); the direct effect represents the effect unexplained by the mediator.

Based on previous research analyses and our findings ([36,37], Table S3), there is a significant relationship between BMI, diet, and PhenoAgeAccel. Poor diet quality may lead to an increase in BMI, and BMI is positively correlated with PhenoAgeAccel (β = 0.35, 95% CI: 0.32, 0.39). Table S4 presents the results of the analysis regarding the mediation of various macronutrients by BMI in relation to PhenoAgeAccel. In terms of macronutrient intake, we found that BMI partially or fully mediates the association between high-quality carbohydrates, total fat, and PhenoAgeAccel. Specifically, the relationship between BMI-mediated high-quality carbohydrates and PhenoAgeAccel had a β (95% CI) of −0.274 (−0.403, −0.140), accounting for 19.76% of the total effect, indicating that approximately one-fifth of the impact is realized through BMI, with BMI playing a significant negative mediating role. Conversely, the relationship between BMI-mediated total fat and PhenoAgeAccel had a β (95% CI) of 0.012 (0.009, 0.016), representing 30.78% of the total effect, suggesting that about one-third of the impact is achieved through BMI, with BMI serving as a significant positive mediator (Figure 1).

### 3.5. Sensitivity Analysis

In the sensitivity analysis, coincident results were found when stratified by gender, race/ethnicity, PIR, drinking, smoking, physical activity, and BMI. The results showed that low-quality carbohydrates, plant protein, USFAs, and SFAs all had an interaction with physical activity (Figure S1).

## 4. Discussion

PhenoAgeAccel, as a novel measure of biological aging [9], has significant implications for health assessment in humans. Its increase is positively correlated with the rising risk of mortality [38]. However, PhenoAgeAccel is a biomarker-based index that measures the discrepancy between an individual’s physiological age and chronological age, reflecting overall health status, whereas mortality risk is an assessment of the likelihood of death events occurring based on epidemiological data. Our research indicates that the intake of high-quality carbohydrates, in conjunction with total protein consumption, contributes to the attenuation of aging, while total fat intake is associated with accelerated aging. In our examination of the relationship between carbohydrates and phenotypic age acceleration, we identified a nonlinear association between high-quality carbohydrate (HQC) intake and PhenoAgeAccel (Figure S2). The variation in PhenoAgeAccel is not a fixed ratio corresponding to changes in HQC consumption. Initially, PhenoAgeAccel increases, indicating accelerated aging; however, after reaching a certain threshold, it decreases, suggesting a deceleration of aging, before rising again. This variation may be influenced by the intake of specific food sources, such as tomatoes and other vegetables (Table S2), which contain bioactive compounds like lycopene and tomatine that can effectively reduce markers of oxidative stress [39,40,41]. These findings suggest that while the intake of high-quality carbohydrates is associated with a reduction in cardiovascular risk factors, weight loss, and a decreased risk of diabetes and cardiovascular diseases [42,43]—resulting in a lower all-cause mortality risk among participants consuming higher amounts of HQCs [44]—excessive intake can also adversely affect health and aging. Overconsumption of simple sugars may lead to various health issues [45]. Caloric restriction may promote healthy aging by altering gene expression and slowing the aging process [46]. Additionally, several studies support a positive correlation between vegetable intake and telomere length [47,48,49], which is regarded as a biological marker of aging and is associated with a shorter life expectancy [50]. However, alternative viewpoints exist in the literature. Some studies report no association between energy intake, dietary energy density, or macronutrient intake and aging in men [28], while others indicate that carbohydrate intake does not significantly correlate with successful aging [26]. In light of these discrepancies, we propose that various quantitative methods for assessing biological aging can measure different aspects of the aging process. A comprehensive understanding of aging necessitates the consideration of multiple biomarkers. Furthermore, the interplay between different nutrients within the body must be accounted for, recognizing their competitive and complementary relationships. Lastly, the representativeness of the population should be considered to assess the generalizability of these conclusions across broader populations.

Moreover, a considerable body of research indicates that plant-based diets may play a role in delaying the aging process [48,51,52,53,54,55], potentially due to the consumption of antioxidant-rich plant proteins (such as nuts and soy) [56,57], which may help mitigate telomere shortening. This connection aligns with our analysis of the impact of plant protein on PhenoAgeAccel. Choosing a diet rich in plant protein can enhance health outcomes and reduce the risk of age-related diseases [58]. Regarding fat intake, animal studies have reported that dietary fat significantly influences aging compared to other macronutrients [59]. Epidemiological evidence has shown associations of total fat intake and saturated fatty acid (SFA) consumption with shorter telomere length [47]. Furthermore, we observed a positive correlation between SFA intake and PhenoAgeAccel, suggesting an acceleration of the aging process.

Reducing the intake of a serving of low-quality carbohydrates, animal proteins, or SFAs while simultaneously increasing by the same amount the intake of high-quality carbohydrates, plant proteins, or USFAs can contribute to delaying the aging process. Several studies on the effectiveness of macronutrient subtypes in relation to age-related diseases support our preliminary conclusions [55,60,61]. Specifically, the consumption of high-quality carbohydrates is associated with lower mortality rates [44], and substituting plant protein for animal protein can decrease overall mortality and cardiovascular disease-specific mortality [55]. There exists a close relationship between the multidimensional characteristics of nutrient intake and healthy aging [62]. By analyzing the intake of different nutritional components, we recommend considering improvements to daily dietary patterns.

Another important finding from our study is that BMI serves as a significant negative mediator in the relationship between high-quality carbohydrates and PhenoAgeAccel, while it acts as a significant positive mediator in the relationship between total fat and PhenoAgeAccel. Existing evidence suggests a connection between macronutrients and BMI [36,37,63], as well as between PhenoAgeAccel and BMI [13], supporting that BMI may play a mediating role in the association between macronutrients and PhenoAgeAccel. Mechanistically, recent animal studies have demonstrated that lycopene [64] and newly identified acetylated derivatives of tomatoes [65] can inhibit adipocyte accumulation. Additionally, there is reported evidence that fruit and vegetable intake is negatively correlated with body mass index [66]. Previous research has highlighted the lipogenic properties of ω-6 polyunsaturated fatty acids [67]. Collectively, these findings support our conclusion that macronutrient intake can modulate BMI. Furthermore, studies indicate that weight changes are associated with age-related diseases, aging, and overall health [13,68,69,70]. Therefore, preventing obesity and selecting high-quality carbohydrates may be linked to the delay of the aging process. Moreover, an increasing number of studies are combining BMI with waist circumference-based measurements to provide a more comprehensive assessment of health status [71,72]. This suggests that future research could further investigate the associations between dietary choices and body shape indices such as the A Body Shape Index (ABSI) and Hip Index (HI) [73].

To our knowledge, this is the first study to simultaneously consider the relationships between the quality, quantity, and food sources of macronutrients and phenotypic acceleration. However, our study has several limitations. First, measurement errors in dietary reporting are inevitable, which may lead to misestimation of the associations. Second, although multiple confounding factors were included, residual confounding may still exist, as the relationship between nutrients and aging is complex and influenced by certain underlying health conditions and lifestyle factors. Third, the cross-sectional design of this study limits causal inferences.

## 5. Conclusions

Different macronutrient consumption subtypes are related to PhenoAgeAccel, which is partially mediated by BMI, depending on the quality of macronutrients. Replacing low-quality macronutrients with high-quality macronutrients might slow aging.

## Figures and Tables

**Table 1:** Baseline characteristics of participants in NHANES, 2005–2010 (n = 6911).

Characteristics	Total (n = 6911)	PhenoAgeAccel		*p*-Value
		≤0	>0	
Gender, n (%)				0.515
Male	3300 (47.34)	2583 (47.07)	717 (48.71)	
Female	3611 (52.66)	2880 (52.93)	731 (51.29)	
Race/ethnicity, n (%)				**0.012**
Non-Hispanic white	3391 (70.81)	2695 (71.67)	696 (66.36)	
Other	3520 (29.19)	2768 (28.33)	752 (33.64)	
PIR, n (%)				**<0.001**
≤1.0	1228 (12.80)	906 (11.69)	322 (18.64)	
>1.0	5144 (87.20)	4144 (88.31)	1000 (81.36)	
Smoking status, n (%)				**<0.001**
Yes	3227 (47.66)	2430 (45.60)	797 (58.30)	
No	3682 (52.34)	3031 (54.40)	651 (41.70)	
Drinking, n (%)				**0.009**
Yes	1129 (19.44)	952 (20.43)	177 (14.31)	
No	5782 (80.56)	4511 (79.57)	1271 (85.69)	
Whether overweight or not, n (%)				**<0.001**
No	1981 (31.73)	1736 (34.45)	245 (17.39)	
Yes	4851 (68.27)	3690 (65.55)	1161 (82.61)	
Moderate or vigorous exercise, n (%)				**<0.001**
Yes	1036 (20.33)	919 (22.14)	117 (10.95)	
No	5875 (79.67)	4544 (77.86)	1331 (89.05)	
Energy intake (kcal/d), mean (sd)	2053.99 (15.46)	2078.00 (16.40)	2929.91 (31.81)	**<0.001**
Total high-quality carbohydrates, serving/d, mean (sd)	2.14 (0.05)	2.20 (0.05)	1.81 (0.08)	**<0.001**
Whole grain, mean (sd)	0.80 (0.03)	0.83 (0.03)	0.65 (0.04)	**<0.001**
Beans, mean (sd)	0.11 (0.01)	0.11 (0.01)	0.10 (0.01)	**0.005**
Fruit, mean (sd)	0.68 (0.02)	0.69 (0.02)	0.60 (0.03)	**<0.001**
Tomatoes, mean (sd)	0.32 (0.01)	0.33 (0.01)	0.26 (0.01)	**<0.001**
Dark green vegetables, mean (sd)	0.14 (0.01)	0.14 (0.01)	0.12 (0.01)	**0.003**
Other vegetables, mean (sd)	0.09 (0.004)	0.09 (0.004)	0.07 (0.01)	**<0.001**
Total low-quality carbohydrates, serving/d, mean (sd)	23.20 (0.40)	23.29 (0.40)	22.74 (0.85)	0.173
Total animal protein, serving/d, mean (sd)	3.50 (0.04)	3.53 (0.04)	3.38 (0.09)	**0.001**
Total plant protein, serving/d, mean (sd)	7.48 (0.09)	7.63 (0.10)	6.68 (0.17)	**<0.001**
Refined grains, mean (sd)	5.57 (0.07)	5.65 (0.07)	5.13 (0.15)	**<0.001**
Beans, mean (sd)	0.45 (0.02)	0.46 (0.02)	0.39 (0.03)	**0.005**
Nuts, mean (sd)	0.59 (0.02)	0.61 (0.03)	0.48 (0.04)	**<0.001**
Soybeans, mean (sd)	0.07 (0.01)	0.08 (0.01)	0.04 (0.01)	**<0.001**
Total SFAs, g/d, mean (sd)	25.67(0.25)	25.82 (0.26)	24.94 (0.57)	**0.002**
Total USFAs, g/d, mean (sd)	45.22 (0.44)	45.59 (0.45)	43.31 (1.06)	**<0.001**
Total carbohydrates, serving/d, mean (sd)	25.33 (0.37)	25.49 (0.38)	24.54 (0.79)	**0.016**
Total protein, serving/d, mean (sd)	10.98 (0.12)	11.16(0.12)	10.06 (0.24)	**<0.001**
Total fat, g/d, mean (sd)	70.89 (0.65)	71.40 (0.67)	68.25 (1.58)	**<0.001**

PIR, poverty income ratio; SFA, saturated fatty acid; USFA, unsaturated fatty acid.

**Table 2:** Multiple linear regression analysis between total macronutrient consumption and PhenoAgeAccel.

Total Macronutrient Consumption	Q1	Q2	Q3	Q4	*p* for Trend	Continuous	*p* Value
	β (95% CI)	β (95% CI)	β (95% CI)	β (95% CI)		β (95% CI)	
Total carbohydrates							
Model 1	reference	0.33 (−0.37, 1.02)	−0.24 (−0.94, 0.45)	−0.03 (−0.78, 0.73)	0.701	−0.001 (−0.02, 0.02)	0.898
Model 2	reference	0.23 (−0.43, 0.89)	−0.08 (−0.78, 0.62)	−0.15 (−0.99, 0.69)	0.566	0.01 (−0.02, 0.04)	0.476
High-quality carbohydrates							
Model 1	reference	−0.29 (−0.94, 0.36)	**−1.30 (−1.99, −0.61)**	**−1.87 (−2.74, −0.99)**	**<0.001**	**−0.29 (−0.52, −0.06)**	**0.015**
Model 2	reference	0.30 (−0.40, 1.00)	**−0.78 (−1.50, −0.05)**	**−1.01 (−1.91, −0.12)**	**0.005**	−0.11 (−0.35, 0.13)	0.368
Low-quality carbohydrates							
Model 1	reference	−0.16 (−0.83, 0.51)	**−0.65 (−1.27, −0.03)**	−0.50 (−1.21, 0.22)	0.214	−0.01 (−0.03, 0.01)	0.436
Model 2	reference	−0.14 (−0.80, 0.52)	−0.51 (−1.13, 0.10)	−0.59 (−1.42, 0.24)	0.143	0.01 (−0.02, 0.04)	0.696
Total protein							
Model 1	reference	**−1.19 (−1.94, −0.44)**	**−1.65 (−2.53, −0.78)**	**−2.74 (−3.87, −1.61)**	**<0.001**	**−0.25 (−0.34, −0.16)**	**<0.001**
Model 2	reference	**−0.94 (−1.66, −0.23)**	**−1.39 (−2.35, −0.43)**	**−2.00 (−3.16, −0.84)**	**0.001**	**−0.16 (−0.26, −0.06)**	**0.002**
Animal protein							
Model 1	reference	−0.12 (−0.98, 0.75)	−0.03 (−0.78, 0.73)	−0.42 (−1.33, 0.49)	0.313	−0.18 (−0.38, 0.02)	0.074
Model 2	reference	−0.14 (−1.02, 0.74)	−0.06 (−0.84, 0.72)	−0.51 (−1.41, 0.40)	0.199	−0.10 (−0.34, 0.14)	0.400
Plant protein							
Model 1	reference	**−0.78 (−1.50, −0.05)**	**−1.11 (−1.92, −0.30)**	**−1.96 (−2.87, −1.05)**	**<0.001**	**−0.20 (−0.29, −0.10)**	**<0.001**
Model 2	reference	−0.65 (−1.35, 0.05)	**−1.10 (−1.87, −0.32)**	**−1.65 (−2.52, −0.78)**	**<0.001**	**−0.15 (−0.25, −0.05)**	**0.004**
Total fat							
Model 1	reference	0.46 (−0.08, 0.99)	**0.86 (0.15, 1.57)**	0.83 (−0.04, 1.71)	0.093	**0.02 (0.01, 0.03)**	**0.007**
Model 2	reference	0.32 (−0.28, 0.92)	0.75 (−0.05, 1.54)	0.92 (−0.14, 1.98)	0.119	**0.03 (0.01, 0.04)**	**<0.001**
SFAs							
Model 1	reference	0.67 (−0.12, 1.46)	**1.02 (0.11, 1.94)**	**1.54 (0.48, 2.60)**	**0.009**	**0.04 (0.01, 0.08)**	**0.020**
Model 2	reference	0.68 (−0.03, 1.39)	**1.07 (0.23, 1.92)**	**1.77 (0.72, 2.81)**	**0.002**	**0.05 (0.02, 0.09)**	**0.003**
USFAs							
Model 1	reference	−0.26 (−0.99, 0.48)	−0.38 (−1.23, 0.47)	−0.72 (−1.80, 0.36)	0.252	−0.01 (−0.02, 0.01)	0.618
Model 2	reference	−0.35 (−1.00, 0.29)	−0.56 (−1.36, 0.24)	−0.83 (−1.99, 0.33)	0.226	0.01 (−0.02, 0.03)	0.548

Model 1: adjusted for sex and race/ethnicity. Model 2: adjusted for sex, race/ethnicity, PIR, smoking status, drinking alcohol, energy intake, physical activity, and whether overweight or not. PIR, poverty income ratio; SFA, saturated fatty acid; USFA, unsaturated fatty acid; Q, quartile.

**Table 3:** Isocaloric substitution effects.

Isocaloric Substitution Effect	Total
Substitution of low-quality carbohydrates by high-quality carbohydrates	**−0.25 (−0.45, −0.05)**
Substitution of animal protein by plant protein	**−0.17 (−0.34, −0.002)**
Substitution of SFAs by USFAs	**−0.06 (−0.10, −0.02)**

Adjusted for sex, race/ethnicity, PIR, smoking status, drinking alcohol, energy intake, physical activity, and whether overweight or not. PIR, poverty income ratio; SFA, saturated fatty acid; USFA, unsaturated fatty acid.

## Data Availability

The data described in the manuscript, code book, and analytic code will be made publicly and freely available without restriction at https://www.cdc.gov/nchs/nhanes/about_nhanes.htm.

## Supplementary Materials

The following supporting information can be downloaded at: https://www.mdpi.com/article/10.3390/nu16203436/s1; Figure S1: Flow chart of the population included in the final analysis of our study; Figure S2: A restricted cubic spline curve was used to fit the relationship between high-quality carbohydrates and PhenoAgeAccel. Adjusted for sex, race/ethnicity, PIR, smoking status, drinking alcohol, energy intake, physical activity and whether overweight or not. HQC: high-quality carbohydrates; Table S1: Per reference unit of subtypes of carbohydrates and protein; Table S2: Multiple linear regression analysis between categorical consumption of carbohydrate and plant protein and phenolAgeAccel; Table S3: Multiple linear regression analysis between BMI and phenolAgeAccel; Table S4: Association between the total macronutrient consumption and PhenoAgeAccel, mediated by BMI; Table S5: The continuous variables of total high-quality carbohydrates, total low-quality carbohydrates, total animal protein, total plant protein, total saturated fatty acids (SFA), and total unsaturated fatty acids (USFA) were divided into quartiles.

## References

[1] Nations U. World Population Prospects 2019: Highlights. https://www.un.org/en/academic-impact/97-billion-earth-2050-growth-rate-slowing-says-new-un-population-report.

[2] Kennedy B.K., Berger S.L., Brunet A., Campisi J., Cuervo A.M., Epel E.S., Franceschi C., Lithgow G.J., Morimoto R.I., Pessin J.E. (2014). Geroscience: Linking aging to chronic disease. Cell.

[3] Niccoli T., Partridge L. (2012). Ageing as a risk factor for disease. Curr. Biol..

[4] Fitzgerald K.N., Hodges R., Hanes D., Stack E., Cheishvili D., Szyf M., Henkel J., Twedt M.W., Giannopoulou D., Herdell J. (2021). Potential reversal of epigenetic age using a diet and lifestyle intervention: A pilot randomized clinical trial. Aging.

[5] Ferrucci L., Levine M.E., Kuo P.L., Simonsick E.M. (2018). Time and the Metrics of Aging. Circ. Res..

[6] Kubben N., Misteli T. (2017). Shared molecular and cellular mechanisms of premature ageing and ageing-associated diseases. Nat. Rev. Mol. Cell Biol..

[7] Costantino S., Paneni F., Cosentino F. (2016). Ageing, metabolism and cardiovascular disease. J. Physiol..

[8] Finkel D., Whitfield K., McGue M. (1995). Genetic and environmental influences on functional age: A twin study. J. Gerontol. B Psychol. Sci. Soc. Sci..

[9] Liu Z., Kuo P.L., Horvath S., Crimmins E., Ferrucci L., Levine M. (2018). A new aging measure captures morbidity and mortality risk across diverse subpopulations from NHANES IV: A cohort study. PLoS Med..

[10] Chang X., Zhou Y.F., Wang L., Liu J., Yuan J.M., Khor C.C., Heng C.K., Pan A., Koh W.P., Dorajoo R. (2022). Genetic associations with healthy ageing among Chinese adults. NPJ Aging.

[11] Klopack E.T., Carroll J.E., Cole S.W., Seeman T.E., Crimmins E.M. (2022). Lifetime exposure to smoking, epigenetic aging, and morbidity and mortality in older adults. Clin. Epigenetics.

[12] Topiwala A., Taschler B., Ebmeier K.P., Smith S., Zhou H., Levey D.F., Codd V., Samani N.J., Gelernter J., Nichols T.E. (2022). Alcohol consumption and telomere length: Mendelian randomization clarifies alcohol’s effects. Mol. Psychiatry.

[13] Cao X., Yang G., Li X., Fu J., Mohedaner M., Danzengzhuoga, Høj Jørgensen T.S., Agogo G.O., Wang L., Zhang X. (2023). Weight change across adulthood and accelerated biological aging in middle-aged and older adults. Am. J. Clin. Nutr..

[14] Duan H., Pan J., Guo M., Li J., Yu L., Fan L. (2022). Dietary strategies with anti-aging potential: Dietary patterns and supplements. Food Res. Int..

[15] Levine M.E., Suarez J.A., Brandhorst S., Balasubramanian P., Cheng C.W., Madia F., Fontana L., Mirisola M.G., Guevara-Aguirre J., Wan J. (2014). Low protein intake is associated with a major reduction in IGF-1, cancer, and overall mortality in the 65 and younger but not older population. Cell Metab..

[16] Senior A.M., Nakagawa S., Raubenheimer D., Simpson S.J. (2020). Global associations between macronutrient supply and age-specific mortality. Proc. Natl. Acad. Sci. USA.

[17] Assmann K.E., Lassale C., Andreeva V.A., Jeandel C., Hercberg S., Galan P., Kesse-Guyot E. (2015). A Healthy Dietary Pattern at Midlife, Combined with a Regulated Energy Intake, Is Related to Increased Odds for Healthy Aging. J. Nutr..

[18] Shammas M.A. (2011). Telomeres, lifestyle, cancer, and aging. Curr. Opin. Clin. Nutr. Metab. Care.

[19] Mathers J.C. (2015). Impact of nutrition on the ageing process. Br. J. Nutr..

[20] Mirzaei H., Suarez J.A., Longo V.D. (2014). Protein and amino acid restriction, aging and disease: From yeast to humans. Trends Endocrinol. Metab..

[21] Dehghan M., Mente A., Zhang X., Swaminathan S., Li W., Mohan V., Iqbal R., Kumar R., Wentzel-Viljoen E., Rosengren A. (2017). Associations of fats and carbohydrate intake with cardiovascular disease and mortality in 18 countries from five continents (PURE): A prospective cohort study. Lancet.

[22] Mendonça N., Granic A., Hill T.R., Siervo M., Mathers J.C., Kingston A., Jagger C. (2019). Protein Intake and Disability Trajectories in Very Old Adults: The Newcastle 85+ Study. J. Am. Geriatr. Soc..

[23] Schoufour J.D., Franco O.H., Kiefte-de Jong J.C., Trajanoska K., Stricker B., Brusselle G., Rivadeneira F., Lahousse L., Voortman T. (2019). The association between dietary protein intake, energy intake and physical frailty: Results from the Rotterdam Study. Br. J. Nutr..

[24] Beaudart C., Locquet M., Touvier M., Reginster J.Y., Bruyère O. (2019). Association between dietary nutrient intake and sarcopenia in the SarcoPhAge study. Aging Clin. Exp. Res..

[25] Moradell A., Fernández-García Á.I., Navarrete-Villanueva D., Sagarra-Romero L., Gesteiro E., Pérez-Gómez J., Rodríguez-Gómez I., Ara I., Casajús J.A., Vicente-Rodríguez G. (2021). Functional Frailty, Dietary Intake, and Risk of Malnutrition. Are Nutrients Involved in Muscle Synthesis the Key for Frailty Prevention?. Nutrients.

[26] Gopinath B., Flood V.M., Kifley A., Louie J.C., Mitchell P. (2016). Association Between Carbohydrate Nutrition and Successful Aging Over 10 Years. J. Gerontol. A Biol. Sci. Med. Sci..

[27] Verspoor E., Voortman T., van Rooij F.J.A., Rivadeneira F., Franco O.H., Kiefte-de Jong J.C., Schoufour J.D. (2020). Macronutrient intake and frailty: The Rotterdam Study. Eur. J. Nutr..

[28] De-la O.A., Jurado-Fasoli L., Gracia-Marco L., Henriksson P., Castillo M.J., Amaro-Gahete F.J. (2022). Association of Energy and Macronutrients Intake with S-Klotho Plasma Levels in Middle-Aged Sedentary Adults: A Cross-Sectional Study. J. Nutr. Health Aging.

[29] Shan Z., Rehm C.D., Rogers G., Ruan M., Wang D.D., Hu F.B., Mozaffarian D., Zhang F.F., Bhupathiraju S.N. (2019). Trends in Dietary Carbohydrate, Protein, and Fat Intake and Diet Quality Among US Adults, 1999–2016. JAMA.

[30] Wei W., Jiang W., Huang J., Xu J., Wang X., Jiang X., Wang Y., Li G., Sun C., Li Y. (2021). Association of Meal and Snack Patterns with Mortality of All-Cause, Cardiovascular Disease, and Cancer: The US National Health and Nutrition Examination Survey, 2003 to 2014. J. Am. Heart Assoc..

[31] Levine M.E., Lu A.T., Quach A., Chen B.H., Assimes T.L., Bandinelli S., Hou L., Baccarelli A.A., Stewart J.D., Li Y. (2018). An epigenetic biomarker of aging for lifespan and healthspan. Aging.

[32] Mekary R.A., Willett W.C., Hu F.B., Ding E.L. (2009). Isotemporal substitution paradigm for physical activity epidemiology and weight change. Am. J. Epidemiol..

[33] Hou W., Gao J., Jiang W., Wei W., Wu H., Zhang Y., Sun C., Li Y., Han T. (2021). Meal Timing of Subtypes of Macronutrients Consumption with Cardiovascular Diseases: NHANES, 2003 to 2016. J. Clin. Endocrinol. Metab..

[34] Han T., Gao J., Wang L., Li C., Qi L., Sun C., Li Y. (2020). The Association of Energy and Macronutrient Intake at Dinner Versus Breakfast with Disease-Specific and All-Cause Mortality Among People with Diabetes: The U.S. National Health and Nutrition Examination Survey, 2003–2014. Diabetes Care.

[35] Sacks F.M., Lichtenstein A.H., Wu J.H.Y., Appel L.J., Creager M.A., Kris-Etherton P.M., Miller M., Rimm E.B., Rudel L.L., Robinson J.G. (2017). Dietary Fats and Cardiovascular Disease: A Presidential Advisory from the American Heart Association. Circulation.

[36] Xiao Q., Garaulet M., Scheer F. (2019). Meal timing and obesity: Interactions with macronutrient intake and chronotype. Int. J. Obes..

[37] Belfort-DeAguiar R., Seo D. (2018). Food Cues and Obesity: Overpowering Hormones and Energy Balance Regulation. Curr. Obes. Rep..

[38] Wang C., Zhong G., Liu C., Hong S., Guan X., Xiao Y., Fu M., Zhou Y., You Y., Wu T. (2024). DNA methylation aging signatures of multiple metals exposure and their mediation effects in metal-associated mortality: Evidence from the Dongfeng-Tongji cohort study. J. Hazard. Mater..

[39] Chaudhary P., Sharma A., Singh B., Nagpal A.K. (2018). Bioactivities of phytochemicals present in tomato. J. Food Sci. Technol..

[40] Fang E.F., Waltz T.B., Kassahun H., Lu Q., Kerr J.S., Morevati M., Fivenson E.M., Wollman B.N., Marosi K., Wilson M.A. (2017). Tomatidine enhances lifespan and healthspan in C. elegans through mitophagy induction via the SKN-1/Nrf2 pathway. Sci. Rep..

[41] Ratto F., Franchini F., Musicco M., Caruso G., Di Santo S.G. (2022). A narrative review on the potential of tomato and lycopene for the prevention of Alzheimer’s disease and other dementias. Crit. Rev. Food Sci. Nutr..

[42] Sievenpiper J.L. (2020). Low-carbohydrate diets and cardiometabolic health: The importance of carbohydrate quality over quantity. Nutr. Rev..

[43] Reynolds A., Mann J., Cummings J., Winter N., Mete E., Te Morenga L. (2019). Carbohydrate quality and human health: A series of systematic reviews and meta-analyses. Lancet.

[44] Hou W., Han T., Sun X., Chen Y., Xu J., Wang Y., Yang X., Jiang W., Sun C. (2022). Relationship Between Carbohydrate Intake (Quantity, Quality, and Time Eaten) and Mortality (Total, Cardiovascular, and Diabetes): Assessment of 2003–2014 National Health and Nutrition Examination Survey Participants. Diabetes Care.

[45] Lee D., Son H.G., Jung Y., Lee S.V. (2017). The role of dietary carbohydrates in organismal aging. Cell Mol. Life Sci..

[46] Waziry R., Ryan C.P., Corcoran D.L., Huffman K.M., Kobor M.S., Kothari M., Graf G.H., Kraus V.B., Kraus W.E., Lin D.T.S. (2023). Effect of long-term caloric restriction on DNA methylation measures of biological aging in healthy adults from the CALERIE trial. Nat. Aging.

[47] Tucker L.A. (2021). Fruit and Vegetable Intake and Telomere Length in a Random Sample of 5448 U.S. Adults. Nutrients.

[48] Rafie N., Golpour Hamedani S., Barak F., Safavi S.M., Miraghajani M. (2017). Dietary patterns, food groups and telomere length: A systematic review of current studies. Eur. J. Clin. Nutr..

[49] Freitas-Simoes T.M., Ros E., Sala-Vila A. (2016). Nutrients, foods, dietary patterns and telomere length: Update of epidemiological studies and randomized trials. Metabolism.

[50] Aubert G., Lansdorp P.M. (2008). Telomeres and aging. Physiol. Rev..

[51] Crous-Bou M., Molinuevo J.L., Sala-Vila A. (2019). Plant-Rich Dietary Patterns, Plant Foods and Nutrients, and Telomere Length. Adv. Nutr..

[52] Foscolou A., Magriplis E., Tyrovolas S., Chrysohoou C., Sidossis L., Matalas A.L., Rallidis L., Panagiotakos D. (2019). The association of protein and carbohydrate intake with successful aging: A combined analysis of two epidemiological studies. Eur. J. Nutr..

[53] Foscolou A., Critselis E., Tyrovolas S., Chrysohoou C., Naumovski N., Sidossis L.S., Rallidis L., Matalas A.L., Panagiotakos D. (2021). The association of animal and plant protein with successful ageing: A combined analysis of MEDIS and ATTICA epidemiological studies. Public Health Nutr..

[54] Naghshi S., Sadeghi O., Willett W.C., Esmaillzadeh A. (2020). Dietary intake of total, animal, and plant proteins and risk of all cause, cardiovascular, and cancer mortality: Systematic review and dose-response meta-analysis of prospective cohort studies. BMJ.

[55] Zheng J., Zhu T., Yang G., Zhao L., Li F., Park Y.M., Tabung F.K., Steck S.E., Li X., Wang H. (2022). The Isocaloric Substitution of Plant-Based and Animal-Based Protein in Relation to Aging-Related Health Outcomes: A Systematic Review. Nutrients.

[56] de Souza R.G.M., Schincaglia R.M., Pimentel G.D., Mota J.F. (2017). Nuts and Human Health Outcomes: A Systematic Review. Nutrients.

[57] Zhang X., Gao B., Shi H., Slavin M., Huang H., Whent M., Sheng Y., Yu L.L. (2012). Chemical composition of 13 commercial soybean samples and their antioxidant and anti-inflammatory properties. J. Agric. Food Chem..

[58] Xu X., Hu J., Pang X., Wang X., Xu H., Yan X., Zhang J., Pan S., Wei W., Li Y. (2024). Association between plant and animal protein and biological aging: Findings from the UK Biobank. Eur. J. Nutr..

[59] Wu Y., Green C.L., Wang G., Yang D., Li L., Li B., Wang L., Li M., Li J., Xu Y. (2022). Effects of dietary macronutrients on the hepatic transcriptome and serum metabolome in mice. Aging Cell.

[60] Seidelmann S.B., Claggett B., Cheng S., Henglin M., Shah A., Steffen L.M., Folsom A.R., Rimm E.B., Willett W.C., Solomon S.D. (2018). Dietary carbohydrate intake and mortality: A prospective cohort study and meta-analysis. Lancet Public Health.

[61] Hunter J.E., Zhang J., Kris-Etherton P.M. (2010). Cardiovascular disease risk of dietary stearic acid compared with trans, other saturated, and unsaturated fatty acids: A systematic review. Am. J. Clin. Nutr..

[62] Senior A.M., Legault V., Lavoie F.B., Presse N., Gaudreau P., Turcot V., Raubenheimer D., Le Couteur D.G., Simpson S.J., Cohen A.A. (2022). Multidimensional associations between nutrient intake and healthy ageing in humans. BMC Biol..

[63] Merino J., Dashti H.S., Sarnowski C., Lane J.M., Todorov P.V., Udler M.S., Song Y., Wang H., Kim J., Tucker C. (2022). Genetic analysis of dietary intake identifies new loci and functional links with metabolic traits. Nat. Hum. Behav..

[64] Fenni S., Hammou H., Astier J., Bonnet L., Karkeni E., Couturier C., Tourniaire F., Landrier J.F. (2017). Lycopene and tomato powder supplementation similarly inhibit high-fat diet induced obesity, inflammatory response, and associated metabolic disorders. Mol. Nutr. Food Res..

[65] Lee J.H., Cho H.D., Jeong J.H., Lee M.K., Jeong Y.K., Shim K.H., Seo K.I. (2013). New vinegar produced by tomato suppresses adipocyte differentiation and fat accumulation in 3T3-L1 cells and obese rat model. Food Chem..

[66] Heo M., Kim R.S., Wylie-Rosett J., Allison D.B., Heymsfield S.B., Faith M.S. (2011). Inverse association between fruit and vegetable intake and BMI even after controlling for demographic, socioeconomic and lifestyle factors. Obes. Facts.

[67] Muhlhausler B.S., Ailhaud G.P. (2013). Omega-6 polyunsaturated fatty acids and the early origins of obesity. Curr. Opin. Endocrinol. Diabetes Obes..

[68] Chen C., Ye Y., Zhang Y., Pan X.F., Pan A. (2019). Weight change across adulthood in relation to all cause and cause specific mortality: Prospective cohort study. Bmj.

[69] Hayes M., Baxter H., Müller-Nordhorn J., Hohls J.K., Muckelbauer R. (2017). The longitudinal association between weight change and health-related quality of life in adults and children: A systematic review. Obes. Rev..

[70] Liu M., Zhang Z., Zhou C., He P., Zhang Y., Li H., Li Q., Liu C., Wang B., Li J. (2021). Relationship of Weight Change Patterns from Young to Middle Adulthood with Incident Cardiovascular Diseases. J. Clin. Endocrinol. Metab..

[71] Bacopoulou F., Landis G., Rentoumis A., Tsitsika A., Efthymiou V. (2017). Mediterranean diet decreases adolescent waist circumference. Eur. J. Clin. Investig..

[72] Chudek A., Owczarek A.J., Ficek J., Olszanecka-Glinianowicz M., Wieczorowska-Tobis K., Walencka Z., Almgren-Rachtan A., Chudek J. (2021). A Stronger Effect of Body Mass Index and Waist Circumference on the Prevalence of Uncontrolled Hypertension among Caucasian Men than Women. Kidney Blood Press. Res..

[73] Krakauer N.Y., Krakauer J.C. (2022). Diet Composition, Anthropometrics, and Mortality Risk. Int. J. Environ. Res. Public Health.

