# Supplementary material for: BMI Mediates the Association between Macronutrient Subtypes and Phenotypic Age Acceleration
Source: Nutrients. 2024 Oct 10;16(20):3436. doi: 10.3390/nu16203436 (PMC11510402; doi:10.3390/nu16203436)
Supplement: Supplementary file 1 [file nutrients-16-03436-s001.zip › nutrients-3234074-supplementary.pdf]

Supplementary Materials

BMI mediates the association between macronutrient subtypes and phenotypic age acceleration

Appendix 1: Supplementary Methods
Appendix 2: Supplemental Results

Appendix 1

Supplementary methods

Measurements and variables

variables		Labels		
Albumin	Standard Biochemistry Profile (L40 C)	LBDSALSI	Albumin (g/L)	g/L
Creatinine	Standard Biochemistry Profile (L40 C)	LBDCSRSI	Creatinine (umol/L)	Umol/L
Glucose	Plasma Fasting Glucose, Serum C-peptide & Insulin (LAB10AM)	LBDGLUSI	Plasma glucose: SI(mmol/L)	Mmol/L
CRP	C-Reactive Protein (CRP), Bone Alkaline Phosphatase (BAP) & Parathyroid Hormone (PTH)(L11 C)	LBXCRP	C-reactive protein (mg/dL)	mg/dL
Lymphocyte percent	Complete Blood Count with 5-part Differential - Whole Blood (L25 C)	LBXLYPCT	Lymphocyte percent (%)	%
Mean cell volume	Complete Blood Count with 5-part Differential - Whole Blood (L25 C)	LBXMCVSI	Mean cell volume (fL)	fL
Red cell distribution width	Complete Blood Count with 5-part Differential - Whole Blood (L25 C)	LBXRDW	Red cell distribution width (%)	%
Alkaline phosphatase	Standard Biochemistry Profile (L40 C)	LBXSAPSI	Alkaline phosphotase (U/L)	u/L
White blood cell count	Complete Blood Count with 5-part Differential - Whole Blood (L25 C)	LBXWBCSI	White blood cell count: SI	SI(1000 cells/uL)
AGE				

PhenoAgeAccel was calculated by age and nine biomarkers which were shown below:

$$\text{Phenotypic Age} = 141.50 + \frac{ln\{-0.00553 \times ln(1 - mortality\ risk)\}}{0.090165}$$

where $\text{Mortality risk} = 1 - \exp\left(\frac{-1.51714 \times exp\,(xb)}{0.0076927}\right)$

and

$xb = -19.907 - 0.0336 \times \text{albumin} + 0.0095 \times \text{creatinine} + 0.1953 \times \text{glucose} + 0.0954 \times ln\,(\text{C−reactive protein}) - 0.0120 \times \text{lymphocyte percentage} + 0.0268 \times \text{mean cell volume} + 0.3306 \times \text{red blood cell distribution width} + 0.00188 \times$

alkaline phosphatase +0.0554 × white blood cell count + 0.0804 × chronological age

PhenoAgeAccel = Phenotypic Age − Age

Appendix 2

Supplementary Results

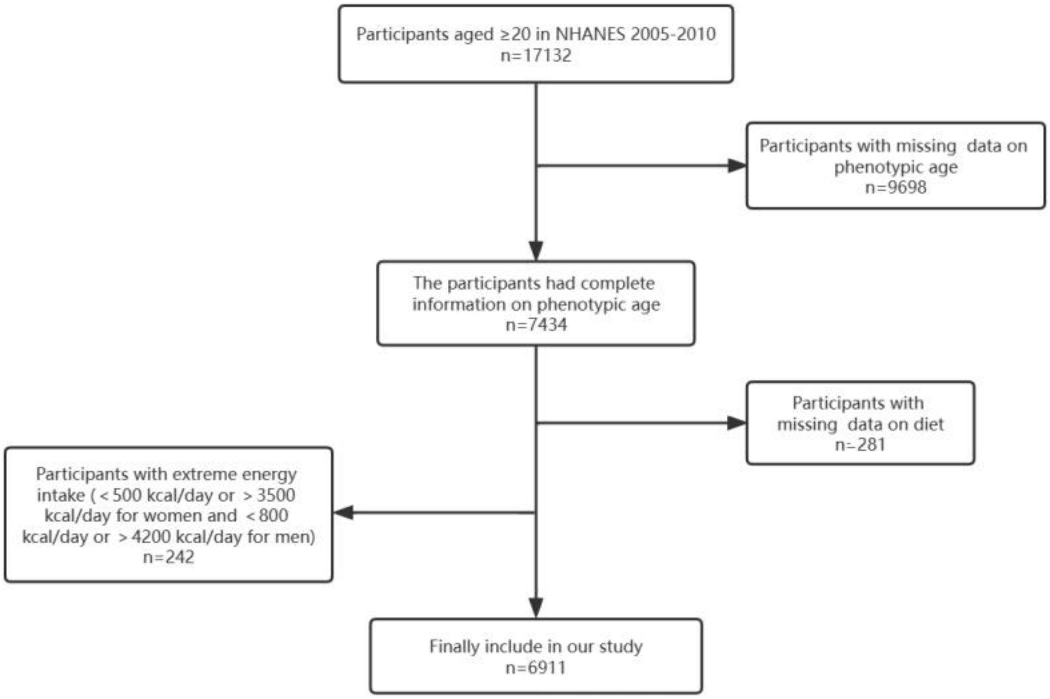

Figure S1. Flow chart of the population included in the final analysis of our study.

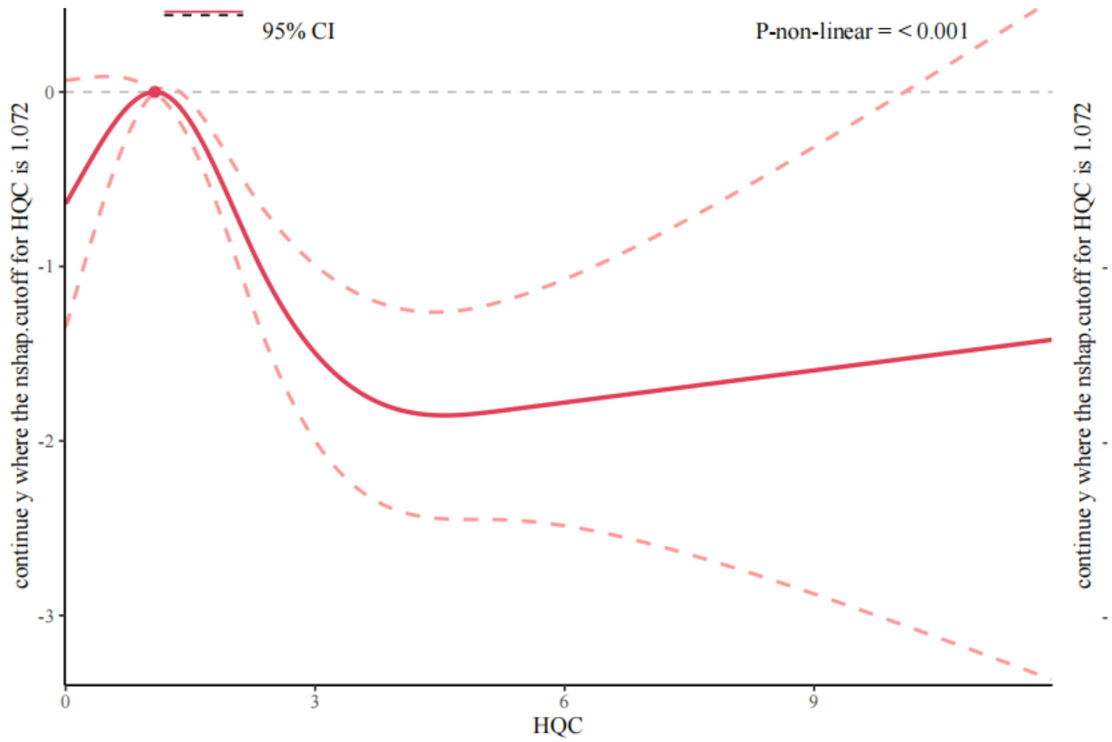

Figure S2. A restricted cubic spline curve was used to fit the relationship between high-quality carbohydrates and PhenoAgeAccel. Adjusted for sex, race/ethnicity, PIR, smoking status, drinking alcohol, energy intake, physical activity and whether overweight or not. HQC: high-quality carbohydrates

Table S1. Per reference unit of subtypes of carbohydrates and protein

Subtypes	Food Patterns Equivalent Database (FPED) Unit representing 1 serving
**High-quality carbohydrates**	
Whole grains	Ounce equivalent
Legumes	Cup equivalent
Intact/whole fruit	Cup equivalent
Tomatoes	Cup equivalent
Dark-green vegetables	Cup equivalent
Other red/orange vegetables	Cup equivalent
**Low-quality carbohydrates**	
Refined grains	Ounce equivalent
Fruit juice	Cup equivalent
Potatoes	Cup equivalent
Other starchy vegetables	Cup equivalent
Added sugars	Teaspoon equivalent
**Animal protein**	
Unprocessed red meat	3.5-ounce equivalents
Processed meat	3.5-ounce equivalents
Poultry	3.5-ounce equivalents
Seafood	3.5-ounce equivalents
Dairy	Cup equivalent
Eggs	Ounce equivalent
**Plant protein**	
Whole grains	Ounce equivalent
Refined grains	Ounce equivalent
Legumes	Cup equivalent
Nuts	Ounce equivalent
Soy	Ounce equivalent

Table S2. Multiple linear regression analysis between categorical consumption of carbohydrate and plant protein and phenolAgeAccel.

Total consumption by macronutrient classification	β (95% CI) Model 1[a]	*P* value	β (95% CI) Model 2[b]	*P* value
High-quality carbohydrates				
Whole grains	-0.26(-0.54, 0.02)	0.066	-0.11 (-0.34, 0.12)	0.333
Legumes	-0.53(-1.46, 0.41)	0.262	-0.35 (-1.38, 0.68)	0.501
Intact/whole fruit	-0.28(-0.59, 0.04)	0.082	-0.13 (-0.42, 0.17)	0.395
Tomatoes	**-1.33(-1.97, -0.70)**	**<0.001**	**-0.93 (-1.56, -0.30)**	**0.005**
Dark-green vegetables	-0.82(-1.88, 0.23)	0.124	-0.32 (-1.28, 0.65)	0.512
Other red/orange vegetables	**-2.54(-3.99, -1.08)**	**0.001**	**-2.36 (-3.70, -1.02)**	**0.001**
Plant protein				
Whole grain	**-0.37(-0.64, -0.09)**	**0.011**	-0.21 (-0.45, 0.26)	0.080
Refined grains	**-0.20(-0.30, -0.10)**	**<0.001**	**-0.19 (-0.30, -0.70)**	**0.002**
Legumes	-0.08(-0.32, 0.16)	0.520	-0.08 (-0.33, 0.17)	0.536
Nuts	**-0.31(-0.46, -0.17)**	**<0.001**	**-0.20 (-0.36, -0.04)**	**0.015**
Soys	**-1.42(-2.18, -0.66)**	**<0.001**	**-1.03 (-1.65, -0.41)**	**0.002**

[a] Adjusted for sex and race/ethnicity.

[b] Adjusted for sex, race/ethnicity, PIR, smoking status, drinking alcohol, energy intake, physical activity, and whether overweight or not.

PIR, poverty income ratio; SFA, saturated fatty acid; USFA, unsaturated fatty acid; Q, quintile

Table S3. Multiple linear regression analysis between BMI and phenolAgeAccel.

	β (95% CI)	*P* value	β (95% CI)	*P* value
	Model 1		Model 2	
BMI				
Continuous	**0.37 (0.33, 0.40)**	**＜ 0.001**	**0.35 (0.32, 0.39)**	**＜ 0.001**
＜ 25	reference		reference	
⩾ 25	**3.42 (3.04, 3.79)**	**＜ 0.001**	**3.17 (2.80, 3.53)**	**＜ 0.001**

Model 1 : Adjusted for sex and race/ethnicity.

Model 2 : Adjusted for sex, race/ethnicity, PIR, smoking status, drinking alcohol, energy intake, and physical activity.

BMI, body mass index; PIR, poverty income ratio

Table S4. Association between the total macronutrient consumption and PhenoAgeAccel, mediated by BMI.

Total macronutrient consumption	β (95% CI)	*P* value
**Total carbohydrates, serving/d**		
Total effect	-0.002 (-0.007, 0.007)	0.872
Direct effect	-0.002 (-0.021, 0.018)	0.869
Mediation effect	-0.000 (-0.007, 0.007)	0.990
Proportion mediated	10.22%	0.791
**Total protein, serving/d**		
Total effect	**-0.193 (-0.254, -0.131)**	**< 0.001**
Direct effect	**-0.180 (-0.235, -0.123)**	**< 0.001**
Mediation effect	-0.013 (-0.036, 0.008)	0.225
Proportion mediated	0.67%	0.225
**Total fat, serving/d**		
Total effect	**0.040 (.029, 0.053)**	**< 0.001**
Direct effect	**0.028 (0.017, 0.040)**	**< 0.001**
Mediation effect	**0.012 (0.009, 0.016)**	**< 0.001**
Proportion mediated	**30.78%**	**< 0.001**
Total high-quality carbohydrates, serving/d		
Total effect	**-0.342 (-0.479, -0.207)**	**< 0.001**
Direct effect	**-0.068 (-0.108, -0.031)**	**< 0.001**
Mediation effect	**-0.274(-0.403, -0.140)**	**< 0.001**
Proportion mediated	**19.76%**	**< 0.001**
Total low-quality carbohydrates, serving/d		
Total effect	-0.005 (-0.029, 0.019)	0.671
Direct effect	-0.007 (-0.030, 0.016)	0.553
Mediation effect	0.002 (-0.005, 0.009)	0.637
Proportion mediated	1.12%	0.981
Total animals protein, serving/d		
Total effect	**0.104 (0.055, 0.152)**	**< 0.001**
Direct effect	-0.039 (-0.192, 0.111)	0.623
Mediation effect	0.065 (-0.094, 0.226)	0.432
Proportion mediated	88.00%	0.432
Total plant protein, serving/d		
Total effect	**-0.171 (-0.235, -0.102)**	**<0.001**
Direct effect	**-0.157(-0.218, -0.092)**	**<0.001**
Mediation effect	-0.014(-0.036, 0.008)	0.224
Proportion mediated	7.75%	0.224
Total SFA, g/d		
Total effect	-0.001 (-0.010, 0.008)	0.858
Direct effect	**0.044 (0.018, 0.071)**	**<0.001**
Mediation effect	**0.043 (0.014, 0.072)**	**<0.001**

Proportion mediated	2.29%	0.844
Total USFA, g/d		
Total effect	**0.020 (0.002, 0.040)**	**0.025**
Direct effect	0.005 (-0.012, 0.023)	0.553
Mediation effect	**0.015 (0.009, 0.020)**	**<0.001**
Proportion mediated	**68.18%**	**0.025**

Adjusted for sex, race/ethnicity, PIR, smoking status, drinking alcohol, energy intake, and physical activity.

Table S5. The continuous variables of total high-quality carbohydrates,total low-quality carbohydrates, total animal protein, total plant protein, total saturated fatty acids (SFA), and total unsaturated fatty acids (USFA) were divided into quartiles.

**Total macronutrient consumption (g)**	**Q1**	**Q2**	**Q3**	**Q4**
**high-quality carbohydrates**	**＜＝0.85**	**＞0.85-＜＝1.70**	**＞1.70-＜＝2.89**	**＞2.89**
**low-quality carbohydrates**	**＜＝12.73**	**＞12.73-＜＝19.63**	**＞19.63-＜＝29.33**	**＞29.33**
**animal protein**	**＜＝2.15**	**＞2.15-＜＝3.06**	**＞3.06-＜＝4.23**	**＞4.23**
**plant protein**	**＜＝4.69**	**＞4.69-＜＝6.80**	**＞6.80-＜＝9.48**	**＞9.48**
**SFA**	**＜＝15.12**	**＞15.12-＜＝22.01**	**＞22.01-＜＝30.81**	**＞30.81**
**USFA**	**＜＝28.06**	**＞28.06-＜＝39.66**	**＞39.66-＜＝54.76**	**＞54.76**

SFA, saturated fatty acids; USFA, unsaturated fatty acids; Q, quintile
